# Supplementary figures and images for: Functional Genomics of a Collection of Gammaproteobacteria Isolated from Antarctica
Source: Mar Drugs. 2024 May 23;22(6):238. doi: 10.3390/md22060238 (PMC11205219; doi:10.3390/md22060238)

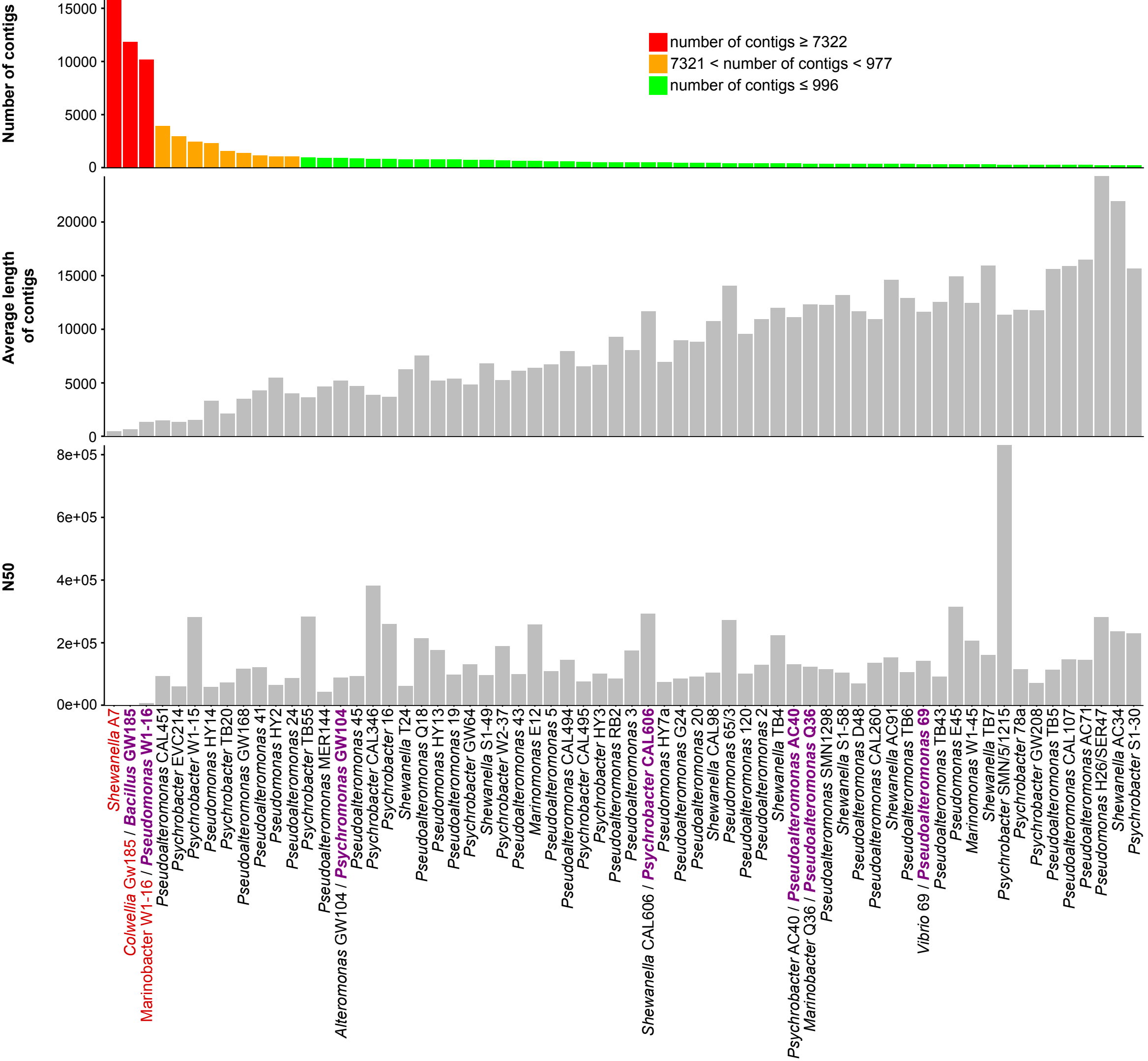

Supplement: Supplementary file 1 [file marinedrugs-22-00238-s001.zip › Supplementary Figure S1.pdf]

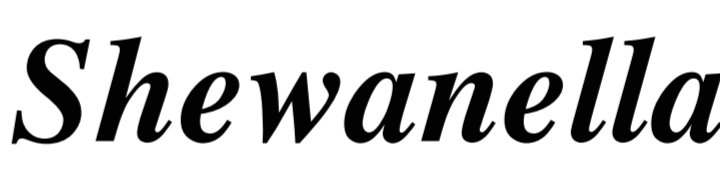

Supplement: Supplementary file 1 [file marinedrugs-22-00238-s001.zip › Supplementary Figure S2.pdf]
